# Description of the cross‐cultural process adopted in the STRiDE (STrengthening Responses to dementia in DEveloping countries) program: A methodological overview

**DOI:** 10.1002/dad2.12293

**Published:** 2022-03-15

**Authors:** Nicolas Farina, Roxanne Jacobs, Tara Puspitarini Sani, Marguerite Schneider, Imelda Theresia, Yuda Turana, Fasihah Irfani Fitri, Emiliano Albanese, Klara Lorenz‐Dant, Sumaiyah Docrat, Petra Du Toit, Cleusa P. Ferri, Ishtar Govia, Adelina Comas‐Herrera, Aliaa Ibnidris, Martin Knapp, Sube Banerjee

**Affiliations:** ^1^ Brighton and Sussex Medical School Brighton UK; ^2^ University of Cape Town South Africa; ^3^ Atma Jaya Catholic University of Indonesia Jakarta Indonesia; ^4^ Alzheimer's Indonesia Indonesia; ^5^ University of Sumatera Utara Indonesia; ^6^ Università della Svizzera Italiana Switzerland; ^7^ The London School of Economics and Political Science UK; ^8^ Alzheimer's South Africa South Africa; ^9^ Universidade Federal de São Paulo Brazil; ^10^ Caribbean Institute for Health Research (CAIHR)—Epidemiology Research Unit The University of the West Indies Jamaica; ^11^ University of Plymouth Plymouth UK

**Keywords:** Afrikaans, Bahasa, cross‐cultural adaptation, dementia, Indonesia, instruments, language, measures, middle income, Sepedi, South Africa, Xhosa

## Abstract

Cross‐cultural adaptation is an important part of using validated questionnaires across countries and settings. Here we describe the cross‐cultural process adopted in the STRiDE (STrengthening Responses to dementia in DEveloping countries) program.

We adopted a cross‐cultural adaptation process including forward translation, back translations, and cognitive interviews of the STRiDE toolkit. In total, 50 older adults and 41 carers across sites in Indonesia and South Africa participated in cognitive interviews; field notes and verbatim quotes are reported.

We describe the cross‐cultural adaptation process of the STRiDE toolkit. During the process, issues were identified with the translated toolkit, including aspects related to cultural appropriateness, terminology equivalence, and timings.

The data demonstrate that a rigorous, yet pragmatic, cross‐cultural adaptation process can be achieved even with limited resources. Our process should help the design and conduct of future dementia research in various contexts.

## 1 INTRODUCTION

Selecting which measures to use across countries is a challenge for global research. The use of standardized and validated measures is essential to allow researchers to measure parameters of interest and to compare findings internally within the study, between countries, and externally with other studies. However, measures developed, standardized, and validated in one setting should be adapted and used cautiously in other settings and countries. As a first step, providing a translation of the measure would seem to be the solution. However, direct translations from one language to another do not guarantee cross‐cultural validity of instruments and measures.^1^ This is particularly problematic in circumstances where direct literal translations occur, resulting in change of the underlying meaning. For example, despite *shame* being the closest translation to *vergüenza* (Spanish), the two are associated with different underlying features (*shame–*moral transgression, humiliation, guilt, wrongdoing, regret, and so on; *vergüenza–*blush, ridicule, shyness, reluctant, and so on).^2^ Direct literal translation alone may also result in terminology that is not culturally appropriate. For example, Wee et al. found that the term *generalized sadness* was more culturally appropriate than *depression* in the Singapore Malay version of the EQ‐5D.^3^

Cross‐cultural adaptation is a “process that looks at both language (translation) and cultural adaptation issues in the process of preparing a questionnaire for use in another setting.”^1^ It has been suggested that cultural adaptation is an important process any time a measure is being used in a different (cultural) population.^4^ There is no gold standard method for cross‐cultural adaptation, which must ensure that the instrument is culturally meaningful while maintaining its content validity.^5^ Multi‐step processes vary mainly in terms of which people are involved in the cross‐cultural adaptation process and the number of unique steps.^5^, ^6^, ^7^ A review of 31 cross‐cultural adaptation guidelines found substantial heterogeneity in the methods used, and a lack of comparison between guidelines.^8^

Cross‐cultural studies in the field of dementia can contribute to advancing knowledge on cohort, temporal, and geographic variations in dementia occurrence, impact, and risks. However, within international dementia research, many studies use instruments developed for use in the United States/Europe, but provide little detail about the language of the measure, the cross‐cultural adaptation process used (if any), or the extent to which the measure has been developed for use within their specific settings.^9^, ^10^, ^11^

This article describes a pragmatic cross‐cultural adaptation process used for the development of a valid and culture‐fair toolkit to enable the collection of data on the prevalence of dementia, its impact on people's lives, and costs in Indonesia and South Africa, as part of the STrengthening Responses to dementia in DEveloping countries (STRiDE, www.stride‐dementia.org/) program. Here we summarize the features of the toolkit that required action and the benefits of adopting such a process, and we provide guidance to replicate it.

## 2 METHODS

### 2.1 The STRiDE toolkit

Few low‐ and middle‐income countries (LMICs) have data on dementia prevalence obtained directly from their own populations. One element of the STRiDE programme aims to fill this gap by generating new prevalence evidence in two (South Africa and Indonesia) of the seven STRiDE countries (Brazil, India, Indonesia, Jamaica, Kenya, Mexico, and South Africa).

**RESEARCH IN CONTEXT**

1. **Systematic Review**: We reviewed the literature using PubMed to identify cross‐cultural adaptation processes used in the field of dementia in low‐ and middle‐income countries. Although the cross‐cultural adaptation process of individual measures is often described, international dementia surveys (composed of multiple questionnaires) often do not describe this process.
2. **Interpretation**: Our findings highlight the importance of a clear and transparent cross‐cultural adaptation process for international dementia research. The process revealed issues that needed to be addressed prior to testing, most notably with terminology and time to complete the survey.
3. **Future Directions**: Although not a definitive cross‐cultural process because adaptation should be driven to accommodate the needs of specific projects, our process should help the design and conduct of future dementia research in various contexts.

We have reported information about STRiDE and the choice of countries elsewhere^12^ and, following this review process, South Africa and Indonesia were selected to capture novel data on the prevalence, costs, and impact of dementia. Both countries were identified as having a need for high‐quality prevalence data.^12^ Preliminary evidence from local studies suggest that 352,000 people have dementia in South Africa,^13^ and over 1.1 million in Indonesia,^14^ highlighting the estimated size of the problem within the countries. Based on the proposed fieldwork methodology, we selected the following settings and languages:

- Semi‐rural Limpopo, South Africa—Sepedi
- Metropolitan Western Cape, South Africa—Afrikaans, isiXhosa, English
- Metropolitan Jakarta, Indonesia—Bahasa Indonesia
- Urban and rural North Sumatra, Indonesia—Bahasa Indonesia

The STRiDE toolkit was developed using a systematic, transparent, and iterative process. This process involved regular iterative meetings and is summarized below:

1. Key outcome domains selected.
2. Summary of candidate measures presented (including their validity and previous use).
3. Identification of country‐specific measures from Indonesia and South Africa teams.
4. Shortlisting and review of hypothetical timings to ensure a fit within an estimated 90‐minute time budget.
5. Two additional meetings to consolidate and refine measures. Cognitive ability measures were compared to existing healthy cognitive aging studies.
6. Consultation with the chief investigator of Alzheimer's Disease International's 10/66 research group (https://1066.alz.co.uk/).

### 2.2 Ethics

We obtained ethical approvals through London School of Economics and Political Science, alongside approvals from the University of Cape Town and Atma Jaya Catholic University of Indonesia.

### 2.3 Procedures

#### 2.3.1 Cross‐cultural adaptation process

We broadly followed the ISPOR Principles of Good Practice and the World Health Organization recommendations for the cross‐cultural adaptation of patient report outcomes.^5^, ^7^ This included:

1. Two translators, proficient in English and experts in the target language, independently performed the forward‐translations of the English toolkit into the target language.
2. The two forward‐translations were then compared and synthesized into a single translation. Discrepancies were discussed between at least one of the forward‐translators alongside two researchers familiar with the original questionnaire but not involved in the translation process. One of these researchers was from the country in which the translation occurred.
3. The final synthesized forward‐translation was then independently back‐translated by two additional translators not involved in the forward‐translation of the toolkit. These translators were required to be proficient in both the target language and English.
4. The two back‐translated English language toolkits were then synthesized into a single translation. Again, discrepancies between translations were discussed in a group that included at least two independent members of the research team and one translator.
5. Inconsistencies between the original toolkit and the synthesized toolkit were then checked. At each stage the emphasis was to retain the original meaning.
6. Lateral comparisons were made between different language translations to ensure consistency in the decision‐making process.
7. Different translations of the toolkit then were used for a series of cognitive interviews with participants that represented the target language and population (described below). This process allowed for a further refinement of the translations based on issues that arose during the testing.
8. Final discussion occurred with local research teams and an independent researcher about the choice of wording and any fine‐tuning of the toolkit.

#### 2.3.2 Participants

A pragmatic recruitment strategy was adopted to identify older adults and carers living in the target areas of the STRiDE prevalence study (i.e., Limpopo, Western Cape, Jakarta, and North Sumatra). Broadly, participants were recruited based on existing contacts, community groups, snowballing, or self‐referrals. However, there were slight variations in recruitment strategy based on setting. Older adults did not need to have a dementia diagnosis or cognitive impairment, although their participation was not discouraged, so long as they had the capacity to consent to participate in the study. The older adult and carer were not required to be participant dyads. Carers were also not limited to individuals caring for persons living with dementia but included any person caring for someone with a chronic illness or disability.

*South Africa*: The identification of English‐, Afrikaans‐, and isiXhosa‐speaking participants (Western cape province) was supported by Dementia‐South Africa (Dementia‐SA), Alzheimer's South Africa (ASA), as well as flyers posted on local WhatsApp community safety forum groups and snowballing techniques. For the Sepedi toolkit, participants were recruited through a social worker from ASA, who arranged access to a seniors’ luncheon club in the Mankweng area (Limpopo province).

*Indonesia*: Participants were identified using an existing list of older adults who had consented previously to be contacted about future research projects. Participants were notified about the research project and asked to contact the research team if interested. Potential participants were also identified from outpatient clinics; they were approached and given the opportunity to participate in the research.

#### 2.3.3 Cognitive interview procedure

We obtained informed consent from all participants. The adapted and translated toolkits (Bahasa Indonesia, Afrikaans, isiXhosa, and Sepedi) alongside English (for South Africa) were then tested in cognitive interviews. Researchers used probes^15^ to better understand why participants responded to the question in a certain way, and to identify questions that participants had difficulty responding to. We adopted a pragmatic approach to identify which items required probing, including:

- where there was difficulty translating the question during the translation process;
- items on which participants were hesitant in their response, or were unable to answer; and
- items that were identified as being particularly associated with culture or country context.

The toolkit was split into an older adult and informant (i.e., carer) toolkit. Researchers initially asked each component of the toolkit in full, without probing. This was to help researchers understand the length of time it took to complete the questionnaires, and provided an opportunity for the researcher and participant to reflect on the responses. The researcher then returned to probe specific questions, making notes of participants’ responses.

### 2.4 Toolkit measures

#### 2.4.1 Older adult and informant questionnaires

- The **Client Service Receipt Inventory (CSRI)** ^16^, ^17^, ^18^ is the main measure of costs and service use. This measure encapsulates items derived from the original CSRI, but also items from the 10/66 household survey and other background information. This included: participant characteristics, household spending, social assistance, social networks, use of services, and care provision.
- The **Community Screening Interview for Dementia** (**CSID)** ^19^ is composed of two components, one a cognitive test for the older person, and the second the informant CSID. The cognitive test assesses language, memory, attention/calculation, orientation, and praxis. The informant CSID captures proxy rating of the target older person's cognitive and functional impairment. The CSID is central to the ascertainment of cases of dementia and, therefore, the prevalence estimate.
- The **World Health Organization Disability Assessment Schedule 2.0 (WHODAS 2.0)** was developed as a measure of disability and functional impairment.^20^ The original 36‐item version of the WHODAS 2.0 was assessed as being too long for the purpose of STRiDE, and so the 12‐item version was selected.
- The **Washington Group Short Set of Questions on Disability (WG‐SS)** ^21^ is a measure that captures disability across a number of universal activities (walking, seeing, hearing, cognition, self‐care, and communication).
- The self‐report **EQ‐5D‐5L** ^22^ is a widely used instrument of generic health‐related quality of life (mobility, self‐care, usual activities, pain/discomfort, and anxiety/depression).
- **Stigma questionnaire**: a questionnaire developed to capture similar concepts as the World Alzheimer's Report Stigma survey,^23^ allowing for future comparisons with worldwide data. The questionnaire captures knowledge, attitudes‐prejudice, and behavior/intentions.

#### 2.4.2 Informant only questionnaires

- The **Dementia Severity Rating Scale (DSRS)** ^24^ is a 12‐item informant‐based, multiple‐choice questionnaire that assesses severity from the mildest to the most severe stages. The DSRS is able to categorize individuals into mild, moderate, and severe groups.
- **Observable Social Cognition: A Rating Scale (OSCARS)** ^25^ is an observer‐based measure of social cognition. Only three items were extracted pertaining to the underlying factor “social cognitive ability.”
- The **Neuropsychiatric Inventory Questionnaire (NPI‐Q)** ^26^ is a shorter version of the NPI‐12 questionnaire, capturing all the same neuropsychiatric domains as the NPI‐12, but focusing only on severity (not frequency).
- **Caregiver Abuse Screen (CASE)** ^27^ is an eight‐item questionnaire developed to identify potential abusive carers. The questions are directed at the carer, and are framed in a manner that does not imply blame or elicit confrontation. The CASE is a tool that can differentiate between abusive and non‐abusive carers.
- The **Zarit Burden Inventory Short Form (ZBI‐12)** ^28^ is a 12‐item version of the 22‐item ZCBI that captures carer burden.
- The **Lawton Instrumental Activities Daily Living Scale (Lawton IADL)** ^29^ captures participant ability to perform eight selected activities including the ability to use the telephone, shop, prepare food, do housekeeping, laundry, transport, and the ability to handle finances and responsibility for own medication.

#### 2.4.3 Older adult–only questionnaires

- Four items from the **Geriatric Mental Schedule (GMS)** ^30^ related to subjective memory complaints. Within the 10/66 study, these four items have been used in algorithm to identify possible mild cognitive impairment.^31^
- The **EURO‐D** ^32^ is a 12‐item scale screening tool for depression. The EURO‐D has two underlying factors: affective suffering and motivation.
- The **Elder Abuse Screening Tool (EAST)** developed as a collaboration between the South African Department of Health and WHO in 2008.^33^ Only the 12‐item self‐report component was used.
- **DEMQOL** ^34^ is a 28‐item validated measure of dementia‐specific health‐related quality of life. The questionnaire is composed of five domains (cognition, negative emotion, positive emotion, social relationships, and loneliness).
- The **Mini‐Cog** ^35^, ^36^ is a three‐item cognitive test that is unaffected by education or language and can be scored by untrained researchers.

#### 2.4.4 Additional measures

- **Translator characteristics**: We captured characteristics about the translators engaged in the translation process.
- **Fieldworker notes**: The researchers made notes in relation to their probes, and general observations during testing. The researchers captured verbatim quotes when relevant.
- **Completion time**: The researchers kept a record of the length of time to complete each questionnaire before probing.

### 2.5 Analysis

We collated descriptive data on the demographic profile of the participants and the translators (Table 1). The analyses of the data were qualitative in nature; fieldworker notes and research team observations were summarized and narratively grouped into key themes. These themes included: non‐equivalent terminology; timings; response formats; and cultural appropriateness. Anonymized *verbatim* quotes from the participant were provided to support the narrative. Responses to the questionnaires were not formally analyzed because this study was of the cross‐cultural adaptation of the instruments rather than a quantitative analysis of the psychometric properties of the instruments produced. The study reported here was not designed or powered to carry out any such analyses. However, such analyses will be completed in the fieldwork stage of STRiDE.

**TABLE 1:** Basic demographics for participants involved in cognitive interviews

	Indonesia	South Africa			
	Bahasa	Sepedi	Afrikaans	English	isiXhosa
Older adult (n)^a^	17	14	6	5	10
Age range (mean; SD)	60‐ 86 (73.57; 6.24)	66‐90 (75.0; 8)	63‐74 (66.5; 4)	53‐78 (68.0; 9)	67‐80 (72.1; 4)
Gender	11F; 1 M	12F; 2 M	3F; 3 M	3F; 2 M	7F; 3 M
Carers (n)	16	10	5	6	7
Age range (mean; SD)	35‐78 (52.33; 16.29)	35‐67 (48.4; 15)	56‐68 (61.3; 6)	42‐73 (54.8; 15)	46‐78 (59.9; 10)
Gender	15F; 1 M	9F; 1 M	5F; 0 M	4F; 1 M	5F; 2 M

Abbreviations: F, Female; M, Male; SD, standard deviation.

^a^The total number of participants (n) involved in the interviews, including those that had partial missing demographic data.

### 2.6 Translator characteristics

See Appendix A for translator characteristics.

## 3 RESULTS

Cognitive interviews were completed with 50 older adults and 41 carers across Indonesia and South Africa. The samples were predominantly female. See Table 1 for participant demographics.

### 3.1 Non‐equivalent terminology

There were instances where there was non‐equivalent terminology within the target language. For example, the term for "privacy" in isiXhosa (*Emfihlakalweni*) is not typically used in daily conversations, whereas the term for "depression" in isiXhosa (*Uxinzelelo lwengqondo*) is used interchangeably with "stress" (*Uxinzelelo*). As such, a direct translation of these words would have led to potential ambiguity in participants’ responses or would capture different concepts than originally intended. During the cognitive interview, researchers described what was meant by the terminology to participants and asked whether there were better terms to use. No single term was identified to accurately represent these concepts in isiXhosa; however, the English words were understood and used.

### 3.2 Examples from specific measures

**CSRI**: Several questions within the CSRI ask about the “head of household.” Although there was already a clear definition of what is defined as “household” (all those that share from the same pot), it was unclear whether the “head” of the household would be the main decisionmaker, the oldest family member (typically male), or the person who earns the most money. For STRiDE we ultimately decided to adopt the definition “the main decisionmaker,” which was more culturally appropriate across settings.

**Stigma questionnaire**: Within the stigma questionnaire, participants were asked whether they have heard of Alzheimer's disease or dementia. For Indonesian participants, it was common for participants to have heard of neither of these words (*penyakit Alzheimer's* and *Demensia*).

**CSID**: In North Sumatera, participants were confused with the word for hammer (*palu*) and were more used to the word *martil*.

### 3.3 Timings

A key issue highlighted was the length of measures. Within South Africa, it was apparent that the length of the toolkit was potentially problematic. For the older adult questions, the length of time to complete the questionnaire (excluding cognitive interview questions) ranged from 97 to 257 minutes; the informant interview completion time ranged from 80 to 268 minutes. In Indonesia, completion times were less problematic, with the older adult interviews lasting between 50 and 167 minutes, and between 52 and 97 minutes for informants.

### 3.4 Response format

In Indonesia, researchers noted that some participants struggled with Likert scale responses. There was a tendency for participants to avoid extreme responses. Within the South African context, Likert scales were considered problematic because participants would forget the response options. Prompt cards were seen as a useful solution to this.

### 3.5 Cultural appropriateness

#### 3.5.1 Examples from specific measures

**CASE and EAST**: Measures of elder abuse were seen to be problematic within Indonesia, particularly when completing the toolkit in a small home setting in which a family member could be overheard. Elder abuse was seen as being taboo within the country, and there were concerns about continued engagement in the broader research if these questions were asked.

**CSID**: Within the CSID, there are several tasks for which the participant has to name objects, including desk, door, and shoes. Within Indonesia, there were instances in which the room or home did not have such items. Substitute items were therefore considered (e.g., instead of pointing to a desk, point to a chair), as well as providing pictorial images as backup (e.g., shoes). Another cognitive question asked about the seasons. While in South Africa, date references are typically used for the four seasons, Indonesia has no official start and end dates of their two seasons (rainy and dry).

## 4 DISCUSSION

The cross‐cultural adaptation process was successful in ensuring that measures that we use for STRiDE are standardized and culturally appropriate. The process revealed issues that needed to be addressed prior to testing, most notably with terminology and time to complete the survey. Issues surrounding a lack of clarity of language used still occurred during cognitive testing, even after rigorous forward‐ and back‐translation, indicating the importance of each step. At times, this was due to differences between colloquial language and slightly more formal language, which meant more formal translations were not always easily understood. Spending additional time talking through the toolkit with the local fieldworkers during the cognitive interview phase was seen of great value to the research team.

Outside of translation, cognitive interviews highlighted potential issues that might affect the practicality of administering the STRiDE toolkit. For example, within Indonesia there was a tendency for participants to avoid extreme responses: this is sometimes referred to as neutral response bias. Such a bias could be considered a "cultural artifact."^37^ One theory is that collectivistic cultures are more concerned about how they fit, compared to individualistic cultures that seek to self‐express and self‐enhance individualistic behaviors and traits.^38^ This has been used to explain why persons socialized in collectivistic cultures such as Malaysia, Indonesia, and the Philippines are more likely to score closely to the neutral mid‐point of a self‐esteem questionnaire compared to those from individualistic countries.^39^

Through the cognitive interviews we were able also to highlight issues with timings. The toolkit length did not result in participant withdrawal. However, the longer testing times meant that visits had to be split over several occasions. Shorter questionnaire length in other studies has demonstrated a 10% increase in participation rate.^40^ However, small differences between questionnaire length may not have much impact on missing data, particularly when response rates are very high.^41^ Inevitably, shorter questionnaires are seen to be the better choice.^42^ To address this, we reduced the length of non‐standardized questionnaires, and through the use of conditional branching (e.g., only people who identify themselves as carers will be asked about burden). As such, only a subset will be asked to complete every single question.

The STRiDE cross‐cultural adaptation process adheres to many of the principles of WHO and IPSOR guidelines, although there are several key differences. First, unlike the WHO cross‐cultural adaptation process, we adopted two independent forward‐ and back‐translation processes, similar to those described elsewhere^1^ and recommended by ISPOR guidelines.^5^ Second, the team that was involved in the reconciliation and synthesis process was not composed of a comprehensive group of experts (e.g., no statistician). Instead, we had a more pragmatic group of individuals comprising research team members. As highlighted elsewhere,^8^ it is unclear if these differences would significantly affect the cross‐cultural adaptation process.

A key challenge was that the process was resource‐intensive, which has been highlighted previously.^4^ Within STRiDE, the translations were carried out by members of the research team and wider colleagues, thus reducing external costs. To reduce costs further we could have removed the back‐translation component and replaced it with a panel of experts (i.e., bilingual, familiar with both cultures, expert in the content measured on the instrument) to review the translations and adaptations, as suggested elsewhere.^43^ To date, there does not appear to be a cost‐free process to translate questionnaires. The use of free e‐translation tools such as “Google Translate,” for example, results in significantly more translation errors than manual translation.^44^

Although outside the scope of this paper, it is important to acknowledge that we did not carry out formal quantitative psychometric evaluation of the instruments within each country setting. This will be completed in the next fieldwork stage of the project. The work presented here is, however, an important first stage in generating instruments that have the necessary statistical validity.

## 5 CONCLUSION

This article describes the cross‐cultural adaptation process used in the STRiDE project, and the potential benefits of adopting such a process for future research. Although the methods described here should not be considered a definitive process or the gold standard, they do provide a template for future pragmatic cross‐cultural dementia research, particularly in LMICs. Table 2 provides a list of recommendations that might assist future cross‐cultural dementia research. It is hoped that the STRiDE cross‐cultural adaptation process can inform and streamline future dementia research in LMICs.

**TABLE 2:** A list of practical recommendations for cross‐cultural adaptation

Ensure that you have a dedicated budget for the time and resources needed to cross‐culturally adapt measures prior to data collection. Specify a systematic process of selecting and cross‐culturally adapting measures from the outset. Clearly describe and report your cross‐cultural adaptation process so that others can understand the process and appraise it. Map existing measures related to your target‐domains. Identify where and how they have been cross‐culturally adapted and validated. Consider whether this evidence is sufficiently robust. Appraise the sensitivity and specificity of your toolkit selection against your study objectives. Consider how the length and types of questions will affect participation and engagement. Involve people outside the immediate research team to minimize potential bias in the cross‐cultural adaptation process. Involve the end‐users (both participants and researchers) in trialing the toolkit. Use cognitive interviews to help understand the thought process underlying their responses.

## FUNDING INFORMATION

This work was supported by the UK Research and Innovation's Global Challenges Research Fund (ES/P010938/1).

## CONFLICTS OF INTEREST

There are no conflicts of interest to declare.

## Supporting information



Supporting information.
