# Supplementary material for: Description of the cross‐cultural process adopted in the STRiDE (STrengthening Responses to dementia in DEveloping countries) program: A methodological overview
Source: Alzheimers Dement (Amst). 2022 Mar 15;14(1):e12293. doi: 10.1002/dad2.12293 (PMC8923343; doi:10.1002/dad2.12293)
Supplement: Supplementary file 1 — Supporting information. [file DAD2-14-e12293-s001.docx]

**Appendix A: Translator characteristics**

All 19 translators (13 South Africa, 6 Indonesia), involved in either the forward- or back-translation of the toolkit, spoke the target language as a first language, and English fluently as a second language. The translators were students (n=7), healthcare professionals (n=3), academic researchers (n=3), research assistants (n=2) and a variety of other professions related to academia, healthcare or translating (n=4). All but two of the translators had previous medical or research questionnaire translation experience, and all but one had experience with medical terminology. All translators had experience with quantitative outcome measures. Past experience in quantitative methods and translator experience was not statistically different between the two countries (p=0.28 and p=0.35, respectively). However, South African translators tended to have more self-reported experience with medical terminology (Median = 3.0, IQR = 2.0), than translators from Indonesia (Median = 1.5, IQR = 1.25) (H=5.52, p=0.02).
